# Clinical features and outcomes of gastric neuroendocrine tumors after endoscopic diagnosis and treatment: A Digestive Endoscopy Society of Tawian (DEST) multicenter study: Erratum

**DOI:** 10.1097/MD.0000000000013472

**Published:** 2018-11-21

In the article, Clinical features and outcomes of gastric neuroendocrine tumors after endoscopic diagnosis and treatment: A Digestive Endoscopy Society of Tawian (DEST) multicenter study”,^[1]^ which appeared in Volume 97, Issue 38 of *Medicine*, the title should be “Clinical features and outcomes of gastric neuroendocrine tumors after endoscopic diagnosis and treatment: A Digestive Endoscopy Society of Taiwan (DEST) multicenter study.”
